# Prenatal exposure of ethanol induces increased glutamatergic neuronal differentiation of neural progenitor cells

**DOI:** 10.1186/1423-0127-17-85

**Published:** 2010-11-12

**Authors:** Ki Chan Kim, Hyo Sang Go, Hae Rang Bak, Chang Soon Choi, Inha Choi, Pitna Kim, Seol-Heui Han, So Min Han, Chan Young Shin, Kwang Ho Ko

**Affiliations:** 1 Department of Pharmacology, College of Pharmacy, Seoul National University, Seoul, Korea; 2 School of Medicine and Center for Neuroscience Research, IBST, Konkuk University, Korea

## Abstract

**Background:**

Prenatal ethanol exposure during pregnancy induces a spectrum of mental and physical disorders called fetal alcohol spectrum disorder (FASD). The central nervous system is the main organ influenced by FASD, and neurological symptoms include mental retardation, learning abnormalities, hyperactivity and seizure susceptibility in childhood along with the microcephaly. In this study, we examined whether ethanol exposure adversely affects the proliferation of NPC and de-regulates the normal ratio between glutamatergic and GABAergic neuronal differentiation using primary neural progenitor culture (NPC) and *in vivo *FASD models.

**Methods:**

Neural progenitor cells were cultured from E14 embryo brain of Sprague-Dawley rat. Pregnant mice and rats were treated with ethanol (2 or 4 g/kg/day) diluted with normal saline from E7 to E16 for *in vivo *FASD animal models. Expression level of proteins was investigated by western blot analysis and immunocytochemical assays. MTT was used for cell viability. Proliferative activity of NPCs was identified by BrdU incorporation, immunocytochemistry and FACS analysis.

**Results:**

Reduced proliferation of NPCs by ethanol was demonstrated using BrdU incorporation, immunocytochemistry and FACS analysis. In addition, ethanol induced the imbalance between glutamatergic and GABAergic neuronal differentiation *via *transient increase in the expression of Pax6, Ngn2 and NeuroD with concomitant decrease in the expression of Mash1. Similar pattern of expression of those transcription factors was observed using an *in vivo *model of FASD as well as the increased expression of PSD-95 and decreased expression of GAD67.

**Conclusions:**

These results suggest that ethanol induces hyper-differentiation of glutamatergic neuron through Pax6 pathway, which may underlie the hyper-excitability phenotype such as hyperactivity or seizure susceptibility in FASD patients.

## Background

Fetal alcohol spectrum disorder (FASD) is a spectrum of mental and physical disorders associated with prenatal exposure to alcohol during pregnancy, which affects one in every 100 live births in United states and Europe [1]. Ethanol has well-known teratogenic effects by mechanisms including induction of apoptosis and inhibition of proliferation, migration, differentiation, and other cellular functions during developmental period [2-5]. In addition, ethanol exposure influences membrane-associated receptor signaling pathways [6], cell adhesion [7,8], and the binding of transcription factors [9].

The central nervous system is the main organ affected by FAS [10-13], and neurological symptoms include mental retardation, learning disabilities and ADHD-like symptoms such as hyperactivity in childhood [14,15]. Children with FASD usually exhibit smaller brain size, so-called microcephaly [16]. Recent studies suggest that alcohol interferes with the migration and organization of brain cells which may cause structural deformities or deficits within the brain.

Neural stem/progenitor cells (NPCs) are self-renewable cells in the CNS. NPC is able to differentiate into specific cell types including neuron during the brain developmental period by its multi-potent capacity. Disorder of neural development might be induced by the de-regulation of NPC proliferation and differentiation, which may cause bigger influence in the entire architecture of the brain compared with the neurotoxic effects of risk factors in later period of life. This is especially true considering the fact that neuron is amitotic after differentiation [17], although there are a few known exceptions [18]. Therefore it is reasonable idea that prenatal ethanol affects overall architecture and size of the brain by influencing the proliferation and differentiation properties of NPCs during developmental periods. Regarding the effect of ethanol on NPCs, it inhibits the proliferation of adult hematopoietic stem cells as well as NPCs [19,20] and suppresses neurogenesis [21,22] in adolescent and adult brain. However, relatively few things are known regarding the effect of ethanol consumption during gestational periods on NPC proliferation and differentiation.

In addition to the regulation of proliferation of NPCs, balance between excitatory and inhibitory neurons in the brain plays a very important role in neurological function of brain. For example, imbalance between excitatory and inhibitory synapses is related to autistic symptoms [23]. This imbalance of excitation and inhibition could be due to the increased excitatory signaling, or to a reduction in inhibition due to a reduction in inhibitory signaling [24]. Increasing the numerical or functional balance of excitatory vs. inhibitory cells can lead to a hyper-excitable state, which might be an underlying neurobiological feature in the manifestation of neurological abnormalities such as hyperactivity symptoms of FASD.

Excitatory neuronal differentiation from NPC is activated by expression of specific transcription factors which act as proneural genes. Proneural genes are both necessary and sufficient to initiate the development of neuronal lineages and to promote the generation of progenitor cells that have a capacity to differentiate. Importantly, proneural genes have been shown to have information into the neurogenesis [25] and to contribute to the control of progenitor-cell identity [26]. Current studies focus on understanding the mechanisms of the multiple functions of proneural genes in neural development [27]. For example, Pax6, a proneural gene originally implicated in eye development, has been suggested in the regulation of glutamatergic neuronal fate. Pax6 induces expression of Ngn2 and NeuroD, which are involved in glutamatergic differentiation and reduces expression of Mash1, which induces GABAergic differentiation.

In this study, we examined the effect of prenatal ethanol consumption on proliferation of NPCs along with the regulation of excitatory and inhibitory neuronal differentiation.

## Methods

### Materials

Hanks balanced salt solution (HBSS), Dulbecco's Modified Eagle's medium/F12 (DMEM/F12), fetal bovine serum (FBS), penicillin/Streptomycin, and 0.25% Trypsin-EDTA were purchased from GibcoBRL (Grand Island, NY). poly-l-ornithine, Tween^® ^20 were purchased from Sigma (St. Louis, MO). ECL™ Western blotting detection reagents were obtained from Amersham Life Science (Arlington Heights, IL). B-27 supplement were purchased from Invitrogen (Carlsbad, CA).

Antibodies were purchased from the following companies: anti-β-actin from Sigma (St. Louis, MO), phospho histone H3 antibody from Upstate Biologicals (Lake Placid, NY), neuronal class III β- tubulin (Tuj-1) antibody from Covance (Richmond, CA), antibodies against nestin, synaptophysin, neuN, Pax6, Neurogenin2 (ngn2) and GAD67 from Millipore (Temecula, CA) and antibodies against Mash1/Achaete-scute homolog 1(Mash1), PSD95, NeuroD1, vGluT1, PCNA and BrdU were obtained from Abcam (Cambrigeshire, England).

### Culture of primary neural stem cells

Neural progenitor cell culture was prepared form E14 embryo SD rat according to previously published procedure [28,29], which was slightly modified by us [30]. In brief, cortices were dissociated into single cells by pipetting several times and passed through 40 μm cell strainer (BD falcon, BD science, Franklin Lakes, NJ). Dissociated single cells were incubated with Dulbecco's modified Eagle's medium/F12 (DMEM/F12) containing B-27 supplement with 20 ng/ml EGF (Upstate) and 10 ng/ml FGF (Invitrogen) at 37°C for 4 days in 5% CO_2 _incubator. The cells grew into floating neurosphere were dissociated with trypsin-EDTA (GibcoBRL) and then resulting single cells were counted and plated on poly-l-ornithine (Sigma) coated plate with DMEM/F12 media containing B-27 supplement for further experiments.

### In vivo ethanol treatment

Pregnant mice and rats were obtained from Daehan Bio Link (Daejeon, Korea) at gestation day (E2) and stabilized under environmental controlled rearing system maintained 12 hr light-dark cycle for 4 days. The animals were treated with ethanol (Hayman, UK; 2 or 4 g/kg/day; 25 v/v %) diluted with normal saline from E7 to E16 via intragastric intubation. Control groups were treated with normal saline. The daily dose was delivered in two halves each in the morning and evening to minimize the deleterious effects of binge alcohol drinking. At E12, P3 and 6 weeks after birth, brain was removed from the offsprings and analyzed for target protein expression by Western blot or immunohistochemistry. All animal experiments were conducted in accordance with the approved procedure either by the Konkuk University or Seoul National University Animal Care and Experimentation Committee.

### Western blot analysis

Cells were washed twice with PBS and lysed with 2× SDS-PAGE sample buffer. An aliquot containing 50 μg of total protein was separated by 10% SDS-PAGE and transferred to nitrocellulose membranes. The membranes were blocked with 1% polyvinylalcohol in PBS containing 0.2% tween-20 for 10 min. The membranes were incubated at 4°C for overnight with first antibodies directed against target proteins such as nestin, tuj-1, pax6, ngn2, neuroD, mash1, PSD95, GAD67(all 1:5000), which were diluted in blocking buffer (5% or 1% skim milk in PBS-Tween (0.2% tween-20)). Membranes were washed 3 times with PBS-Tween for 10 min, and then incubated with species specific peroxidase-conjugated secondary antibodies (Santa Cruz, CA), which were diluted in blocking buffer (5% skim milk in PBS-Tween) for 2 hrs at room temperature. Specific bands were detected using the ECL system (Amersham) and exposed to Bio-Rad electrophoresis image analyzer (Bio-Rad, Hemel Hampstead, UK).

### MTT assay

To determine the viability of cell, we used MTT assay. NPCs were incubated for 60 min with 500 μg/ml MTT reagent (3-(4, 5-dimethylthiazol-2-yl)-2,5-diphenyltetrazlium bromide, a tetrazole, Sigma) in the dark. After incubation, medium was removed and the formazan dye was extracted using 100% ethanol. The absorbance was determined using a microplate reader (Spectrafluor, Tecan Trading AG, Austria) at 590 nm.

### BrdU (5-bromo-2-deoxyuridine, Bromodeoxyuridine) incorporation

Proliferation of NPCs was measured using BrdU ELISA kit (Roche, Mannheim, Germany) following manufacturer's instruction. After ethanol treatment, cells grown in 96-well plate were incubated at 37°C for 24 hrs with 10 μM of BrdU labeling solution. After removing BrdU labeling solution, cells were fixed for 30 min at room temperature. Fixative was washed away and 100 μl of anti-BrdU solution was added for 2 hrs. After washing with PBS for three times, colors were developed using anti-BrdU-POD solution and were incubated for 10-30 min at room temperature. We added 1N HCl (50 μl/well) until the absorbance was sufficient for photometric detection and then the absorbance was measured using an ELISA reader (Spectrafluor) at 450 nm.

### Fluorescent Activated Cell Sorting Analysis (FACS)

Cell cycle of NPCs was analyzed by FACS analysis. Plated single cells were trypsinized with trypsin-EDTA and were suspended in PBS with 1% FBS. Suspension was centrifuged at 3000 rpm for 3 min and supernatant was removed as completely as possible without disturbing the pellet. Suspended cell was fixed with 70% ethanol in PBS and was incubated for overnight at 4°C. Supernatants were removed after centrifugation as above and cells were incubated with 50 μg/ml propidium iodide (Sigma) and 100 μg/ml ribonuclease A (Sigma) in 500 μl PBS with 1% FBS. Samples were kept at room temperature, protected from the light for 30-40 min prior to analysis. Cell cycle of NPCs was analyzed using an FACS cytometer (BD bioscience).

### Immunocytochemistry

Cultured NPCs or differentiated cells on cover glass (Fisher Scientific, PA) were washed and fixed with 4% paraformaldehyde at 4°C for 2 hrs. The cells were treated with 0.3% Triton X-100 for 15 min at room temperature and were blocked for 30 min with blocking buffer (1% BSA, 5% FBS in PBS) at room temperature. The cells were incubated for overnight at 4°C with primary antibodies against phospho-histone H3 (rabbit, 1:500), tuj-1 (rabbit, 1:500), nestin (mouse, 1:500), GAD67 (mouse, 1:500), and neuroD (rabbit, 1:500) diluted in blocking buffer, and were washed with washing buffer (0.1% BSA, 0.5% FBS in PBS) for 3 times. Secondary antibodies conjugated with TMRE (anti-mouse, 1:100) or FITC (anti-rabbit, 1:100 were diluted in blocking buffer and incubated for 2 hrs at room temperature in the dark condition.), In some cases, nucleus was co-stained with DAPI (4'-6-diamidino-2-phenylindole) staining solution (1:100, Invitrogen). After washed 3 times with washing buffer, the cover glass were mounted in Vectashield (Vector laboratories, Burlingame, CA) and viewed with a confocal microscope (TCS-SP, Leica, Heidelberg, Germany).

### Statistical analysis

Data were expressed as the mean ± standard error of mean (S.E.M) and analyzed for statistical significance using one way analysis of variance (ANOVA) followed by Newman-Keuls test as a post hoc test and a P value < 0.05 was considered significant.

## Results

### Ethanol inhibited proliferation of neural stem cell

**Figure 1:**
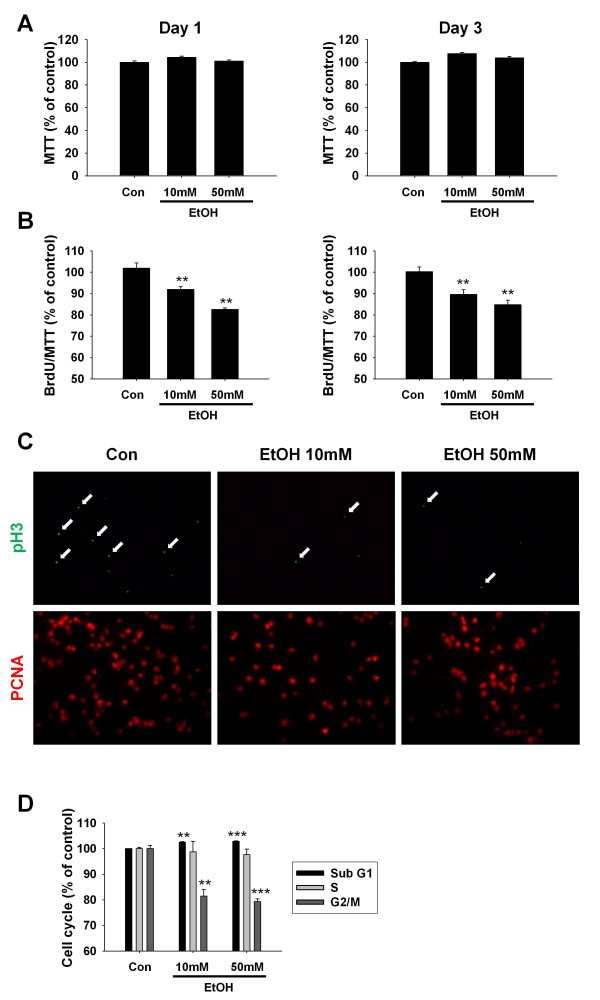
**Ethanol inhibited the proliferation of NPCs**. We treated two concentrations (10 mM and 50 mM) of ethanol to rat primary NPCs culture for 1 or 3 days. Cell viability (A) and BrdU incorporation (B) was examined as described in methods. (A) MTT analysis. Ethanol did not induce cellular toxicity against NPCs. (B) Both on day 1 and 3, BrdU incorporation was inhibited by ethanol treatment in a concentration-dependent manner. (C) To investigate inhibitory effect of ethanol on cell proliferation, immunocytochemistry against pH3 or PCNA was performed on day 3. The number of pH3-positive cells as well as PCNA positive cells was reduced by ethanol treatment. (D) FACS analysis of cell cycle. FACS analysis was performed as described in methods 4 hr after ethanol treatment on NPCs culture. Ethanol treatment decreased cells in G2/M phase as compared with control. Values are expressed as the mean ± S.E.M. **, *** *p *< 0.01 and < 0.001 vs. control (n = 5 for A, B and C. n = 3 for D).

We first determined the effect of ethanol on NPCs viability. Ethanol did not show toxicity to NPCs culture, which was determined by MTT assay at all concentration and duration we used in this study (Figure 1A). To determine anti-proliferative effect of ethanol, BrdU incorporation assay was performed. BrdU is a synthetic nucleoside that is an analogue of thymidine, which is commonly used for the detection of proliferating cell. The BrdU assay measures cells that have synthesized DNA within a given time period. The percentage of BrdU-positive cells was reduced compared with control after treatment with 10 and 50 mM ethanol (Figure 1B). The inhibition of BrdU incorporation by ethanol showed concentration dependency and the extent of inhibition was higher when the cells were treated with ethanol for 3 days.

To further investigate the anti-proliferative effect of ethanol, cells were immunostained for phospho-histone H3 (pH3) and Proliferating Cell Nuclear Antigen (PCNA), as markers for dividing cells. The number of pH3 or PCNA-positive cell was significantly reduced by ethanol treatment in a concentration dependent manner (Figure 1C) suggesting that ethanol inhibits the cell cycle progression of NPCs culture.

To determined mechanism of anti-proliferative effect of ethanol, we performed FACS analysis. Quantitative graph represented relative proportion of sub G1, S and G2/M phases in control and 10 or 50 mM ethanol treated groups. In quantitative analysis of FACS data, ethanol treatment to NPCs culture slightly increased cells in sub G1 phase and decreased the proportion of cells in G2/M phase as compared with control (Figure 1D) suggesting the inhibitory role of ethanol during G2/M cell cycle progression of NPCs culture.

### Ethanol increased neurogenesis

**Figure 2:**
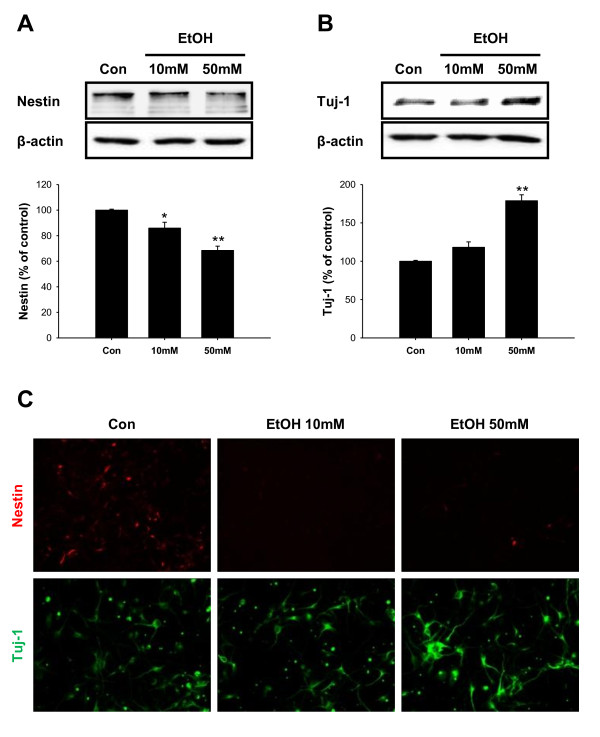
**Ethanol induced early neurogenesis from NPCs**. (A) Expression of Nestin and (B) Tuj-1 was determined by Western blot after ethanol treatment. Ethanol (50 mM) decreased the expression of Nestin to 70% of control level and increased that of Tuj-1 to 170% of control value. (C) Immunocytochemical staining of nestin and Tuj-1. Similar results were obtained as Western blot. Values are expressed as the mean ± S.E.M. *, ** *p *< 0.05 and < 0.01 vs. control (n = 5).

We next examined the differentiation of NPCs by Western blot analysis and immunocytochemistry assays using cell specific marker proteins. Nestin was used as an undifferentiated neural stem cell marker, and Tuj-1 was used for neuron. In western blot analysis, the level of nestin was decreased on day 3 after ethanol treatment (Figure 2A), which is consistent with the inhibitory effect of ethanol on NPCs proliferation as described in Figure 1. On the contrary, the level of Tuj-1 was significantly increased about 2-fold compared to control with 50 mM of ethanol treatment (Figure 2B). These results suggest that ethanol induced neural stem cell differentiation into neuron while inhibiting the proliferation of NPCs in the early stage of neurogenesis. In immunochemical staining, the number of nestin positive cells was decreased by ethanol treatment while Tuj-1 positive cells showed increased number and length of neural processes with stronger immunoreactivity (Figure 2C). The differences in neural differentiation by ethanol were disappeared if we extended the differentiation period to 7 days suggesting that ethanol may promote the kinetics of neural differentiation but not the neural fate (neuron vs. glia) determination itself (data not shown) in our experimental condition.

### Glutamatergic neuronal differentiation was induced by ethanol through Pax6 expression

**Figure 3:**
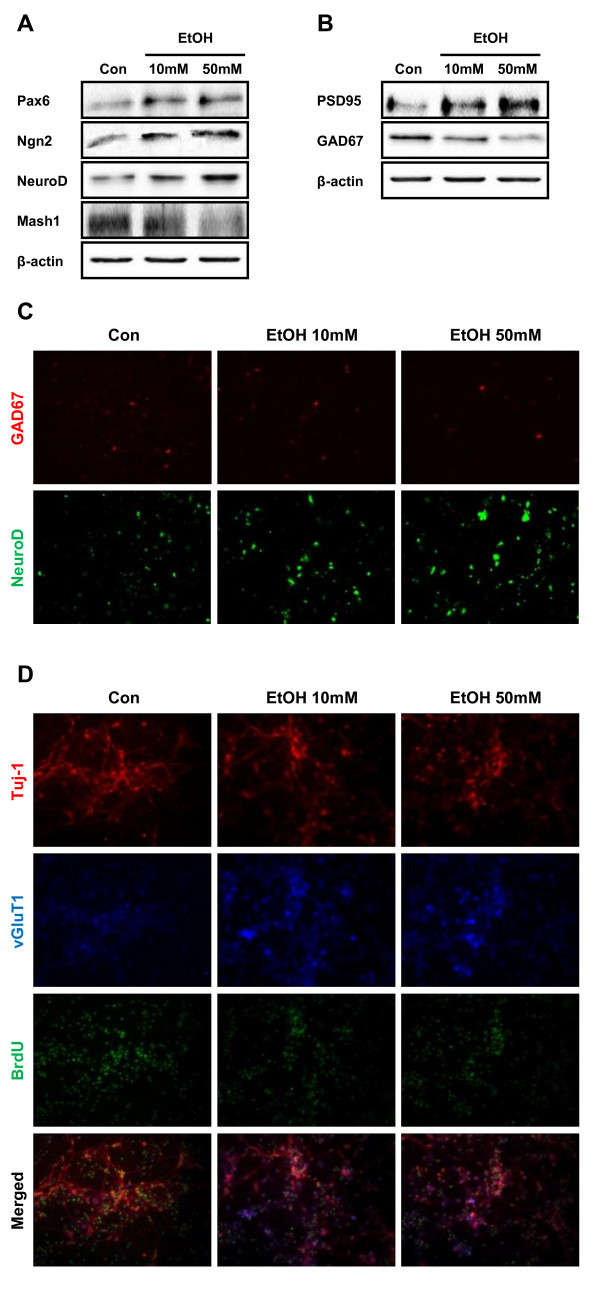
**Increased expression of Pax6 and glutamatergic neuronal differentiation by ethanol treatment**. NPCs were treated with ethanol and Western blot and immunocytochemistry were performed to determine the expression of Pax6 and downstream transcription factors (A) as well as glutamatergic and GABAergic neuronal subtype markers (B). (C) Immunocytochemical staining of GABAergic marker GAD67 and a regulator of excitatory neuronal differentiation, NeuroD, in NPCs treated with ethanol. (D) Triple immunocytochemical staining of neuronal marker Tuj1 (red) and vGluT1 (blue), a marker for glutamatergic neuron along with BrdU (green) staining, a marker for proliferated cells. Most of the vGluT1-positive cells were co-localized with BrdU staining.

To investigate whether ethanol alters the balance of excitatory/inhibitory neuronal differentiation, we first examined the level of expression of proneural genes after ethanol treatment. Proneural genes such as Pax6, Ngn2 and NeuroD are expressed in stepwise pattern during developmental periods and have been suggested to promote excitatory neuronal differentiation. Expression of Pax6, Ngn2 and NeuroD was increased 1 day after ethanol treatment compared to control (Figure 3A). However, the level of Mash1, which have been implicated in inhibitory neuronal differentiation, was decreased in the same condition (Figure 3A). These data suggest that the number of excitatory neuron might be higher than that of inhibitory neuron and we performed Western blot analysis using the marker protein, PSD95 as a glutamatergic neuronal marker and GAD67 as an inhibitory neuronal marker. The level of PSD95 was significantly increased in neurons differentiated for 7 days from NPCs by single ethanol treatment. On the contrary, the level of GAD67 was decreased in the same condition (Figure 3B). Immunocytochemistry also showed increased expression of NeuroD and decreased expression of GAD67 by ethanol treatment (Figure 3C). Immunocytochemical reactivity for vGluT1, a marker for glutamatergic neuron, also increased by ethanol treatment (Figure 3D). Positive cells against vGluT1 were also positive against BrdU staining, suggesting that neural progenitor cells are differentiated into glutamatergic neuron. Altogether, these results suggest that exposure to ethanol induced early neurogenesis while inhibiting proliferation of NPCs, and modified the balance of glutamatergic/GABAergic neuronal differentiation.

### Increased expression of Pax6 and glutamatergic neuronal differentiation by prenatal ethanol exposure in vivo

**Figure 4:**
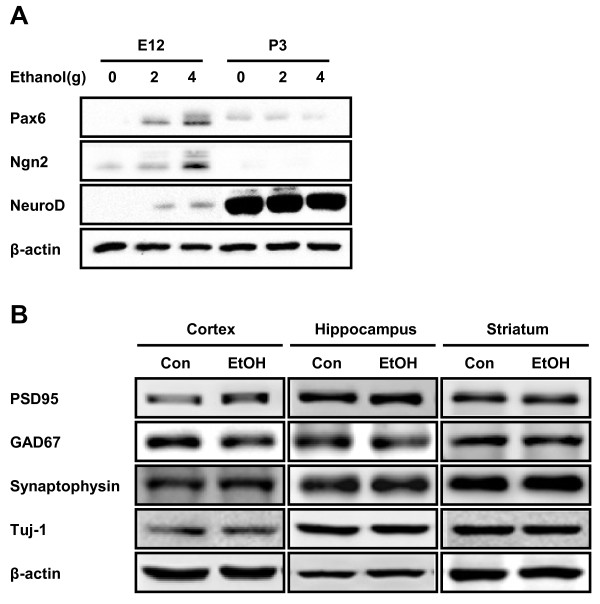
**Increased expression of Pax6 and glutamatergic neuronal differentiation *in vivo *by ethanol treatment**. (A) Expression level of Pax6, Ngn2 and NeuroD was determined by Western blot as described, which showed significant increase during embryonic stage by *in vivo *ethanol treatment in FASD animal model. (B) Expression level of PSD95, GAD67, synaptophysin and Tuj-1 in the 6 week-brain of FASD animal model. Expression of PSD95 was up-regulated in the cortex and striatum. On the contrary, GAD67 expression was decreased in the cortex.

Next, we examined the effect of ethanol on neural stem cell differentiation in FASD animal models. Pregnant mice were administered with ethanol (2 g/kg and 4 g/kg) on E6 until E16 and we investigated the expression of Pax6, Ngn2 and NeuroD by Western blot. The level of these transcription factors was significantly increased in the brain of E12 embryonic mice from dams ingested ethanol (Figure 4A). At postnatal day 3, expression level of Pax6 and Ngn2 was decreased both in control and ethanol groups almost below the detection limit and the level of NeuroD, which modulates neuronal maturation, was significantly increased in postnatal period although there is not much difference between treatment groups (Figure 4A). We next examined the expression level of PSD95, GAD67, synaptophysin and Tuj-1 in the several brain regions of FASD rat animal models at 6 weeks, the time point that the neural developments are already completed. Compared to the control group, the level of PSD95 was significantly increased in cortex and to a lesser extent in hippocampus, but not in striatum. Likewise, we observed a slight increase in the expression level of synaptophysin in cortex and hippocampus of prenatally ethanol exposed rats. On the other hand, the level of GAD67 was reduced in the cortex and hippocampus of prenatally ethanol-treated group. The level of Tuj-1 and β-actin determined by Western blot (Figure 4B) as well as NeuN and Tuj-1 immunohistochemical staining (data not shown) did not show significant difference in all brain regions examined, which suggest that the total number of neuron is not different between control and prenatally ethanol-exposed groups. Altogether, these results suggest that prenatal ethanol exposure induced glutamatergic neuronal differentiation through increased expression of Pax6, Ngn2 and NeuroD in both *in vitro *and *in vivo *conditions.

## Discussion

Excess alcohol consumption during pregnancy exerts teratogenic effects on the fetus, including abnormalities of the central nervous system, general growth retardation and craniofacial defects, which are collectively called FASD [31-35]. Recently, it becomes clear that prenatal exposure to ethanol may induce alterations in neurobehavioral phenotypes or performance of executive functions in the offsprings without obvious physical deformation such as facial changes. It is self-evident that the neuropathological changes may involve either or both the alterations in neural stem cell proliferation and differentiation, and a few studies investigated the effects of prenatal alcohol exposure on the NPCs proliferation and neuronal development. Previous studies have suggested that prenatal ethanol exposure may affect CNS development, which range from the apoptotic death of stem cell population to modulation of cell cycle progress during neurulation or neurogenesis periods [33,36-38]. More recently, it has been suggested that alcohol may affect the differentiation of cortical neurons *in vitro *[37] as well as hippocampal neurons *in vivo *[39]. In addition, alterations in astroglial differentiation have also been suggested [40,41]. Here, we demonstrated that ethanol inhibited proliferation of NPCs and induced early differentiation of neuron. It also modulated excitatory/inhibitory neuronal differentiation both *in vitro *and *in vivo*, which might be related to the hyper-excitability of prenatally ethanol-exposed subjects.

Although increased apoptosis [42], interruption to cell proliferation [43], and impaired protein and DNA synthesis [44] have been reported as a possible mechanism underlying the teratogenic effect of ethanol, mechanisms regulating the neurological symptoms of FASD have not been clearly explained yet. Suggested mechanisms includes DNA methylation [45,46], modulation of phospholipase D signaling [47], apoptosis [48-50], and alteration in neuronal migration [51] as well as changes in neurotransmitter systems [52].

Excitatory neuronal differentiation from NPCs is activated by expression of specific transcription factors. Past studies emphasized the role of Pax6 in eye development [53,54]. Recently, another role of Pax6 as a neuronal subtype determinant is magnified. Pax6 is expressed at NPCs committed to glutamatergic neuronal fate [55]. Pax6 induces the expression of Ngn2 and NeuroD, which again involved in glutamatergic differentiation, while reduces the expression of Mash1, an enhancer of GABAergic differentiation [56-60].

However, it should be remembered that the expression of Pax6 is also associated with the regulation of stem cell proliferation and brain microcephaly. In the neocortex, functional loss of Pax6 results in microcephaly which might be induced by an abnormal development of the secondary progenitor population of the subventricular zone (SVZ), also known as basal progenitor cells (BP cells) [61-64]. In a study using *Xenopus *embryo, Peng et al reported that exposure to ethanol reduced the expression of several regulators of development including *Xenopus *Pax6 (xPAX6) more than 90%, which might be related to the microcephaly [65]. More recently, similar findings were reported with pregnant Wistar rats and their offsprings [66]. Obviously, these results are inconsistent with our results, which showed increase in Pax6 level by ethanol treatment both *in vivo *and *in vitro*. The most important difference of the previous experiments and ours might be the difference in the route of ethanol treatment. In the study of Aronne et al., they treated pregnant Wistar rats with ethanol by intraperitoneal injection (3.5 g/kg) from gestational day 10 to 18 (G10-G18). Interestingly, they found that fetal weights and cerebral cortex thickness were significantly lower in G18 prenatally ethanol exposed rat fetuses than in control fetuses as well as neural tube defects. In our study, we used gastric intubation protocol to mimic actual binge drinking situation and did not found defects in weight gain and any other physical malformations suggesting that our protocol is much milder compared to that of other researchers, although it is also possible that species difference may account for the different results. Whether there is biphasic bell shaped concentration response curve for the expression level of Pax6 and the resulting neurodevelopmental consequences, would be a intriguing and must be answered question to further extend our understanding about the effect of parental alcohol consumption on the neurobiological phenotype in offsprings.

In the present study, prenatal ethanol promoted excitatory neuronal differentiation, possibly via increased expression of Pax6, Ngn2 and NeuroD. Increasing the numerical ratio of excitatory/inhibitory cells can lead to a hyper-excitable state, which might be related to the hyperactivity symptoms observed in FASD patients. In fact, defects in either the production or migration of cortical GABAergic neurons can lead to decreased numbers of cortical GABAergic neurons, which result in a hyper-excitable cortex [67]. Mutations in GAD65, which may also induce the reduction of inhibition in the mouse cerebral cortex, interfere in the maturation of binocular vision [68]. After perinatal early exposure to ethanol, the expression of GABA_A _receptor or GABA synaptic proteins as well as GABAergic synaptic transmission has been reported to be impaired [52,69,70]. Fetal exposure to alcohol is also related to a higher susceptibility to convulsions. Recently, it has been suggested that genetically epilepsy prone rats (GEPRs) display susceptibility to audiogenic seizure after fetal exposure to ethanol while there is general reduction in susceptibility against pentylenetetrazole-induced seizure compared to cognate control [71].

Although the mechanism for molecular signaling pathway directly modulating the ratio of excitatory/inhibitory neuron is unclear yet, the results from the present study may suggest that ethanol modulates the expression of key transcriptional factors involved in the excitatory neuronal differentiation. Whether the modulation of Pax6, Ngn2 and NeuroD by prenatal ethanol treatment is causally related to the regulation of excitatory neuronal differentiation and to hyperactive neuronal phenotype should be investigated further in the future study.

## Conclusions

In this study, we demonstrated that ethanol exposure suppressed the proliferation of NPCs and affected excitatory/inhibitory neuronal subtype differentiation. Decreased proliferation of NPCs by ethanol was identified using BrdU incorporation, pH3 immunostaining and FACS analysis. Ethanol induced glutamatergic neuronal differentiation, possibly via transient increase in the expression of Pax6, Ngn2 and NeuroD with concomitant decrease in the expression of Mash1. Similar pattern of expression of above transcriptional factors as well as glutamatergic neuronal differentiation was shown using *in vivo *model. These results suggest that ethanol-induced hyper-differentiation of glutamatergic neuron via Pax6 pathway may underlie the hyper-excitability phenotype such as hyperactivity or seizure susceptibility in FASD, which may provide additional insights into the understanding of neurological aspects of FASD and devising pharmacological and molecular biological methods leading to the better treatment options.

## Competing interests

The authors declare that they have no competing interests.

## Authors' contributions

KCK participated in study design and conceptualization, analyzed data, and wrote the manuscript. HSG participated in data collection, analysis and study design. HRB performed experiment and helped with composing manuscript. CSC, IC and PK performed experiment for *in vivo *model. S-HH participated in study design. SMH helped with experiment. CYS conceptualized and designed the study. KHK contributed study design and revised the manuscript for intellectual content. All authors read and approved the final manuscript.
